# Examining the environmental risk factors of progressive-onset and relapsing-onset multiple sclerosis: recruitment challenges, potential bias, and statistical strategies

**DOI:** 10.1007/s00415-023-11980-z

**Published:** 2023-09-28

**Authors:** Ying Li, Alice Saul, Bruce Taylor, Anne-Louise Ponsonby, Steve Simpson-Yap, Leigh Blizzard, Simon Broadley, Jeannette Lechner-Scott, Ausimmune/AusLong Investigators Group, Robyn Lucas, Keith Dear, Anne-Louise Ponsonby, Terry Dwyer, Ingrid van der Mei, Leigh Blizzard, Steve Simpson-Yap, Bruce Taylor, Simon Broadley, Trevor Kilpatrick, David Williams, Jeanette Lechner-Scott, Cameron Shaw, Caron Chapman, Alan Coulthard, Michael Pender, Patricia Valery, Rana Karabudak, Francesco Patti, Sara Eichau, Marco Onofrj, Serkan Ozakbas, Dana Horakova, Eva Kubala Havrdova, Francois Grand’Maison, Raed Alroughani, Oliver Gerlach, Maria Pia Amato, Ayse Altintas, Marc Girard, Pierre Duquette, Yolanda Blanco, Cristina Ramo-Tello, Guy Laureys, Tomas Kalincik, Samia J. Khoury, Vahid Shaygannejad, Masoud Etemadifar, Bhim Singhal, Saloua Mrabet, Matteo Foschi, Mario Habek, Nevin John, Stella Hughes, Pamela McCombe, Radek Ampapa, Anneke van der Walt, Helmut Butzkueven, Koen de Gans, Chris McGuigan, Celia Oreja-Guevara, Maria Jose Sa, Thor Petersen, Talal Al-Harbi, Angel Perez Sempere, Bart Van Wijmeersch, Nikolaos Grigoriadis, Julie Prevost, Orla Gray, Tamara Castillo-Triviño, Richard Macdonell, Alessandra Lugaresi, Seyed Aidin Sajedi, MSBase, Rana Karabudak, Francesco Patti, Sara Eichau, Marco Onofrj, Serkan Ozakbas, Dana Horakova, Eva Kubala Havrdova, Francois Grand’Maison, Raed Alroughani, Oliver Gerlach, Maria Pia Amato, Ayse Altintas, Marc Girard, Pierre Duquette, Yolanda Blanco, Cristina Ramo-Tello, Guy Laureys, Tomas Kalincik, Samia J. Khoury, Vahid Shaygannejad, Masoud Etemadifar, Bhim Singhal, Saloua Mrabet, Matteo Foschi, Mario Habek, Nevin John, Stella Hughes, Pamela McCombe, Radek Ampapa, Anneke van der Walt, Helmut Butzkueven, Koen de Gans, Chris McGuigan, Celia Oreja-Guevara, Maria Jose Sa, Thor Petersen, Talal Al-Harbi, Angel Perez Sempere, Bart Van Wijmeersch, Nikolaos Grigoriadis, Julie Prevost, Orla Gray, Tamara Castillo-Triviño, Richard Macdonell, Alessandra Lugaresi, Seyed Aidin Sajedi, Jamie Campbell, Cees Zwanikken, Vincent van Pesch, Guillermo Izquierdo, Davide Maimone, Bianca Weinstock-Guttman, Murat Terzi, Alexandre Prat, Cavit Boz, Magd Zakaria, Liesbeth van Hijfte, Bassem Yamout, Pierre Grammond, Juan Ignacio Rojas, Daniele Spitaleri, Jeannette Lechner-Scott, Katherine Buzzard, Olga Skibina, Nevin Shalaby, Riadh Gouider, Edgardo Cristiano, Jens Kuhle, Mark Slee, Recai Turkoglu, L. G. F. Sinnige, Jose Luis Sanchez-Menoyo, Claudio Solaro, Elisabetta Cartechini, Gerardo Iuliano, Bruce Taylor, Farouk Talaat, Michael Barnett, Jiwon Oh, Maria Edite Rio, Ricardo Fernandez-Bolaños, Dheeraj Khurana, Sarah Besora, Aysun Soysal, Maria Laura Saladino, Leontien Den Braber-Moerland, Jose Antonio Cabrera-Gomez, Barbara Willekens, Justin Garber, Waldemar Brola, Yara Fragoso, Abdullah Al-Asmi, Allan Kermode, Marzena Fabis-Pedrini, Emmanuelle Lapointe, Suzanne Hodgkinson, Claudia Vasconcelos, Patrice Lalive, Cameron Shaw, Claudio Gobbi, Nevin Shalaby, Simon Cardenas-Robledo, Todd Hardy, Elizabeth Alejandra Bacile, Eugenio Pucci, John Parratt, Seyed Mohammad Baghbanian, Carlos Vrech, Deborah Field, Ilya Kister, Jan Schepel, Joyce Pauline Joseph, Melissa Cambron, Norma Deri, Carmen-Adella Sirbu, Fraser Moore, Magda Tsolaki, Mike Boggild, Nai-Wen Tsai, Neil Shuey, Shlomo Flechter, Simu Mihaela, Alejandro Jose Diaz Jimenez, Chu Zhen Quek, Danny Decoo, Dimitrios Karussis, Eduardo Aguera-Morales, Etienne Roullet, Ik Lin Tan, Jabir Alkhaboori, Jihad Inshasi, Karim Kotkata, Katrin Gross-Paju, Magdolna Simo, Mona Al Khawajah, Nazanin Razazian, Stephane Charest, Tunde Csepany, Vetere Santiago, Yaou Liu, Ingrid van der Mei

**Affiliations:** 1 Menzies Institute of Medical Research, University of Tasmania, 17 Liverpool Street, Hobart, TAS 7000 Australia; 2 Florey Institute for Neuroscience, University of Melbourne, Melbourne, VIC Australia; 3 Melbourne School of Population and Global Health, Neuroepidemiology Unit, The University of Melbourne, Melbourne, VIC Australia; 4 School of Medicine and Dentistry, Griffith University, Brisbane, QLD Australia; 5 Hunter Medical Research Institute, University of Newcastle, Callaghan, Australia; 6 Hacettepe University, Ankara, Turkey; 7 Department of Medical and Surgical Sciences and Advanced Technologies, GF Ingrassia, Catania, Italy; 8 UOS Sclerosi Multipla, AOU Policlinico “G Rodloico-San Marco”, University of Catania, Catania, Italy; 9 Hospital Universitario Virgen Macarena, Seville, Spain; 10 University G. d’Annunzio, Chieti, Italy; 11 Dokuz Eylul University, Konak, Izmir Turkey; 12 Charles University in Prague and General University Hospital, Prague, Czech Republic; 13 Neuro Rive-Sud, Quebec, Canada; 14 Amiri Hospital, Sharq, Kuwait; 15 Zuyderland Medical Center, Sittard-Geleen, The Netherlands; 16 School for Mental Health and Neuroscience, Maastricht University, Maastricht, The Netherlands; 17 University of Florence, Florence, Italy; 18 Department of Neurology and Koc University Research Center for Translational Medicine (KUTTAM), Koc University, School of Medicine, Istanbul, Turkey; 19 CHUM and Universite de Montreal, Montreal, Canada; 20 Hospital Clinic de Barcelona, Barcelona, Spain; 21 Hospital Germans Trias i Pujol, Badalona, Spain; 22 Universitary Hospital Ghent, Ghent, Belgium; 23 Department of Neurology, Neroimmunology Centre, Royal Melbourne Hospital, Melbourne, Australia; 24 Department of Medicine, CORe, University of Melbourne, Melbourne, Australia; 25 American University of Beirut Medical Center, Beirut, Lebanon; 26 Isfahan University of Medical Sciences, Isfahan, Iran; 27 Al Zahra Hospital, Isfahan, Iran; 28 Bombay Hospital Institute of Medical Sciences, Mumbai, India; 29 Department of Neurology, LR 18SP03, Clinical Investigation Centre Neurosciences and Mental Health, University Hospital Razi-Manouba, Tunis, Tunisia; 30 Faculty of Medicine of Tunis, University of Tunis El Manar, 1007 Tunis, Tunisia; 31 S. Maria delle Croci Hospital, AUSL Romagna, Ravenna, Italy; 32 Neuroscience Section, Department of Applied Clinical Sciences and Biotechnology, University of L’Aquila, Via Vetoio 1, L’Aquila, Italy; 33 University Hospital Center Zagreb, Zagreb, Croatia; 34 University of Zagreb, School of Medicine, Zagreb, Croatia; 35 Monash Medical Centre, Melbourne, Australia; 36 Department of Medicine, School of Clinical Sciences, Monash University, Melbourne, Australia; 37 Royal Victoria Hospital, Belfast, UK; 38 Royal Brisbane and Women’s Hospital, Brisbane, Australia; 39 University of Queensland, Brisbane, Australia; 40 Nemocnice Jihlava, Jihlava, Czech Republic; 41 The Alfred Hospital, Melbourne, Australia; 42 Central Clinical School, Monash University, Melbourne, Australia; 43 Monash University, Melbourne, Australia; 44 Groene Hart Ziekenhuis, Gouda, Netherlands; 45 St Vincent’s University Hospital, Dublin, Ireland; 46 University College Dublin, Dublin, Ireland; 47 Hospital Clinico San Carlos, Madrid, Spain; 48 Centro Hospitalar Universitario de Sao Joao, Porto, Portugal; 49 Faculty of Health Sciences, University Fernando Pessoa, Porto, Portugal; 50 Aarhus University Hospital, Aarhus, Denmark; 51 King Fahad Specialist Hospital-Dammam, Khobar, Saudi Arabia; 52 Hospital General Universitario de Alicante, Alicante, Spain; 53 Pelt and Hasselt University, Hasselt, Belgium; 54 AHEPA University Hospital, Thessaloniki, Greece; 55 CSSS Saint-Jérôme, Saint–Jerome, Canada; 56 South Eastern HSC Trust, Belfast, UK; 57 Hospital Universitario Donostia and IIS Biodonostia, San Sebastián, Spain; 58 Austin Health, Melbourne, Australia; 59 Department of Medicine, The University of Melbourne, Melbourne, Australia; 60 Florey Institute for Neuroscience, The University of Melbourne, Melbourne, VIC Australia; 61 IRCCS Istituto delle Scienze Neurologiche di Bologna, Bologna, Italy; 62 Dipartimento di Scienze Biomediche e Neuromotorie, Università di Bologna, Bologna, Italy; 63 Golestan University of Medical Sciences, Gogan, Iran

**Keywords:** Progressive-onset multiple sclerosis, Environmental factors, Case–control, Subject recruitment, Bias

## Abstract

It is unknown whether the currently known risk factors of multiple sclerosis reflect the etiology of progressive-onset multiple sclerosis (POMS) as observational studies rarely included analysis by type of onset. We designed a case–control study to examine associations between environmental factors and POMS and compared effect sizes to relapse-onset MS (ROMS), which will offer insights into the etiology of POMS and potentially contribute to prevention and intervention practice. This study utilizes data from the Primary Progressive Multiple Sclerosis (PPMS) Study and the Australian Multi-center Study of Environment and Immune Function (the AusImmune Study). This report outlines the conduct of the PPMS Study, whether the POMS sample is representative, and the planned analysis methods. The study includes 155 POMS, 204 ROMS, and 558 controls. The distributions of the POMS were largely similar to Australian POMS patients in the MSBase Study, with 54.8% female, 85.8% POMS born before 1970, mean age of onset of 41.44 ± 8.38 years old, and 67.1% living between 28.9 and 39.4° S. The POMS were representative of the Australian POMS population. There are some differences between POMS and ROMS/controls (mean age at interview: POMS 55 years vs. controls 40 years; sex: POMS 53% female vs. controls 78% female; location of residence: 14.3% of POMS at a latitude ≤ 28.9°S vs. 32.8% in controls), which will be taken into account in the analysis. We discuss the methodological issues considered in the study design, including prevalence-incidence bias, cohort effects, interview bias and recall bias, and present strategies to account for it. Associations between exposures of interest and POMS/ROMS will be presented in subsequent publications.

**Supplementary Information:**

The online version contains supplementary material available at 10.1007/s00415-023-11980-z.

## Introduction

Multiple sclerosis (MS) is a chronic inflammatory neurodegenerative disorder of the central nervous system that carries a high personal morbidity and economic burden [1]. Clinically, MS can be divided into relapse-onset MS (ROMS) and progressive-onset MS (POMS). ROMS presents with periods of acute neurological impairment (relapse) followed by complete or partial remission, while POMS presents with a progressive phase without relapses or remission from onset[2]. ROMS generally includes people with relapsing–remitting MS (RRMS), of which a majority later convert to the secondary progressive MS (SPMS) phenotype where relapses largely cease and disability progression is more marked, while POMS is synonymous with primary progressive multiple sclerosis (PPMS) as stated in the Lublin classification [2, 3].

There is a consensus that these two MS onset types are not separate diseases but subtypes of the same process, with the observed relatively minor differences in magnetic resonance imaging (MRI) and pathological markers seemingly more quantitative than qualitative [3, 4]. Nonetheless, studies have identified differences between the two groups. For example, in POMS, the female to male sex ratio is much closer to one [5], the mean age at onset is around 10 years later than ROMS [6], and the latitudinal gradient in the frequencies of disease seems merely absent [7, 8]. From a pathophysiological perspective, a diffuse axonal degeneration is observed in POMS, in contrast to the more inflammatory demyelinating lesions seen in ROMS [9]. In recent years, dramatic progress has been made in the development of treatments for patients with relapsing–remitting MS [10, 11], while only one treatment, ocrelizumab, has been shown as effective in reducing the rate of disability progression and MRI changes in people with POMS [12, 13].

Despite the apparent differences in particularly the epidemiology of progressive vs. relapse-onset disease, there has been relatively little work examining POMS cases in isolation. Instead, observational studies examining the environmental risk factors of MS have typically just included all people with MS with a minority of POMS cases, in keeping with their frequencies in the population, roughly 80–85% with ROMS and 10–15% with POMS. Accordingly, unless sample sizes were large, case–control and cohort studies have been unable to conduct analyses by onset type. Therefore, the current established and canonical risk factors for MS may be more likely to reflect the etiology of ROMS rather than that of POMS, and their relevance to POMS needs to be clarified.

Studies focusing specifically on risk factors in samples of POMS are limited, and none examined these characteristics in comparison to a parallel sample of ROMS. A group in Iran conducted two separate case–control studies between 2018 and 2020, including cases who were diagnosed with PPMS during the study period and sex-matched controls who were identified through random-digit dialling. The first study focused on dietary intake during adolescence (*n* = 143 cases, 400 controls) [14], and the second study on stressful life events in the 5 years prior to diagnosis (*n* = 146 cases, 294 controls) [15]. POMS cases were more likely to report lower intakes of dairy, seafood, red meat, vegetables, fruit, and nuts, suggesting that higher intakes of these food groups during adolescence may be associated with a reduced risk of POMS [14]. None of the specific stressful life events in the 5 years prior to diagnosis was associated with an increased risk of POMS [15]. Depression history in the 5 years before POMS diagnosis was also associated with a 3.48-fold higher risk of POMS onset, though this could be interpreted as part of what is now known as an the MS prodrome [16].

It is important to understand whether the current known risk factors for MS also specifically apply to people with POMS and whether the effect sizes are similar for POMS compared to ROMS. It will offer insights in the importance of specific risk factors and their associated mechanisms of action. We conducted a case–control study examining whether the established risk factors for MS also hold in people with POMS, whether the effect sizes are similar or different compared to people with ROMS, and whether there are risk factors for those with POMS that have not previously been shown in ROMS. The study was linked to the AusImmune Study, which has previously implicated or substantiated a number of key risk factors for MS risk, including sun exposure and vitamin D [17, 18], early-life hygiene-related factors [19], occupational exposures [20], offspring number and pregnancy [21], anti-Epstein–Barr virus antibody levels and infectious mononucleosis history [22], smoking [11], and diet [23].

In this publication, we outline the methods and conduct of the Primary Progressive Multiple Sclerosis (PPMS) Study, assess whether the POMS sample is representative, and discuss the methodological issues considered in study design and planned analysis. Descriptive results regarding the samples are presented, whilE associations between exposures of interest and POMS/ROMS will be presented in subsequent publications.

## Methods

### Description of the AusImmune Study

The AusImmune Study is an incident, matched, multi-center case–control study (2003–2006) with participants recruited from four regions of Australia (Fig. 2) [24]. The cases are people aged 18–59 years, with a first clinical diagnosis (FCD) of central nervous system demyelination within the study period, including (1) those with a classic first demyelinating event (FDE, defined as a single, first, episode of demyelination with no recalled prior undiagnosed episodes suggestive of an FDE), (2) those presenting with an FCD who on specific questioning also had an earlier historical episode suggestive of an FCD), and (3) those with a first clinical diagnosis of POMS. Cases were referred to the study by medical specialists after the first clinical presentation and a study neurologist confirmed the date and symptomatology of their FCD and conducted a detailed neurologic examination. Clinical information for each case was reviewed annually by the study neurologist group to assess eligibility. A total of 282 FCD cases were recruited and 279 were included in the analyses.

FCD cases were subsequently followed in the ongoing extension study, the AusImmune Longitudinal (AusLong) Study, to investigate the risk factors for early MS progression through annual telephone data collection, and face-to-face interviews at 2 or 3 years, 5 years, and 10 years post-baseline. A 15-years follow-up is in process. This included the assessment of conversion to MS, based on clinical and/or MRI parameters in keeping with MS diagnostic criteria [25].

The controls for the AusImmune Study were randomly selected from the Australian Electoral Roll, matched on age (within 2 years), sex, and region of residence of the cases. Among the 1118 initially selected controls, 937 (84%) were successfully contacted, and 558 (60%) were matched to an eligible case and included in the study. Based on the estimated incidence of FCD in the source populations, the following case:control ratios were defined for each study region: Brisbane City 1:2, Newcastle Region 1:4, Geelong City and the Western Districts of Victoria 1:3, Tasmania 1:1 [20].

For the current study, we use the data for FCD participants who subsequently developed MS and the control participants. Of the original 282 FCD participants, 3 were excluded during follow-up because their presenting event was found to be due to another neurological condition (one neuromyelitis optica spectrum disorder, one Susac’s syndrome, and one pineal germinoma), and thus not a valid FCD. Of the remaining 279 participants, 236 participated at the 5-years review and 225 at the 10-years review, among which 204 (73.1%) are classified as ROMS and 18 (6.5%) as POMS, whereas 36 (12.9%) did not develop MS and 21 (7.5%) were lost to follow-up (14 no longer wished to participate, 3 relocated and became uncontactable, 2 were too ill to continue participating, and 2 were deceased). These numbers align with the literature, with approximately 65% converting to MS after 10 years [26].

### Study design considerations

The original plan for the PPMS Study was to recruit 350 people with prevalent POMS, aged 18–59 years, and resident in Australia. We included prevalent rather than incident cases, as the incidence of POMS is too low to recruit incident POMS in a logistically feasible fashion. Further, we expanded from the four Australian regions in the AusImmune Study to include recruitment from throughout Australia. With over 25,600 Australians living with MS, of which an estimated 10–15% are POMS, there were estimated to be 2560–3840 POMS cases that potentially could participate in the study. Beyond this initial case–control study, we also planned to conduct a longitudinal study, as was done in the AusLong Study, following the POMS participants annually and examining the factors predicting clinical progression.

An initial target sample size of 350 was selected to provide sufficient statistical power for the examination of interactions between main effects, while allowing for drop-out in the longitudinal study over 5–10 years follow-up in the longitudinal phase. For the longitudinal study, we included those aged ≥ 60 years. Thus, those < 60 years were invited to participate in both the case–control study and the longitudinal study, while those ≥ 60 years were only invited to participate in the longitudinal study.

Unfortunately, recruitment was more challenging than anticipated. This required a number of changes to the case–control study design:

- Reducing the recruitment target to 150 participants: as a result, for a binary exposure of 30%, 40%, 50% prevalence, the minimum OR (for univariate associations between risk factors and the probability of developing PPMS) able to be detected with 80% power is 1.71, 1.68, 1.69 in the smaller sample of 150 cases and 558 controls than 1.50, 1.47, 1.47 in the original sample of 350 cases and 558 controls.
- Expanding the age range to include people over the age of 60 years: we recruited additional participants who agreed to participate in the longitudinal study to also participate in the case–control study. We first invited those aged 60–62 years and gradually increased the cutoff from 60 to 62 to 65 years as required.
- Due to logistical limitations and the high costs involved (using a commercial pathology service), biological sampling was discontinued and only samples from the first 48 participants were retained for future analysis.
- With a dedicated longitudinal study becoming less feasible, it was decided to instead invite participants to become part of the Australian MS Longitudinal Study (AMSLS). The AMSLS is managed by author van der Mei. This study tracks patient-reported outcomes of Australian with MS over time and runs surveys on specific topics at annual or other intervals.

### Case definition and confirmation of eligibility

To recruit participants for both the case–control and longitudinal study, we included participants with POMS who were ≥ 18 years and resident in Australia. The final inclusion criteria of the case–control study included people with POMS, aged 18–65 years at the time of recruitment, and resident in Australia. After informed consent, the participant’s treating neurologist was approached to determine the eligibility for definite primary progressive MS according to the 2010 McDonald criteria [27]. The treating neurologist was also asked to provide an estimated date of first symptom and diagnosis date of MS. The study neurologist (BT) contacted the case and/or physician and reviewed the medical notes as needed to clarify information regarding a formal diagnosis.

### Participant recruitment strategies

**Fig. 1:**
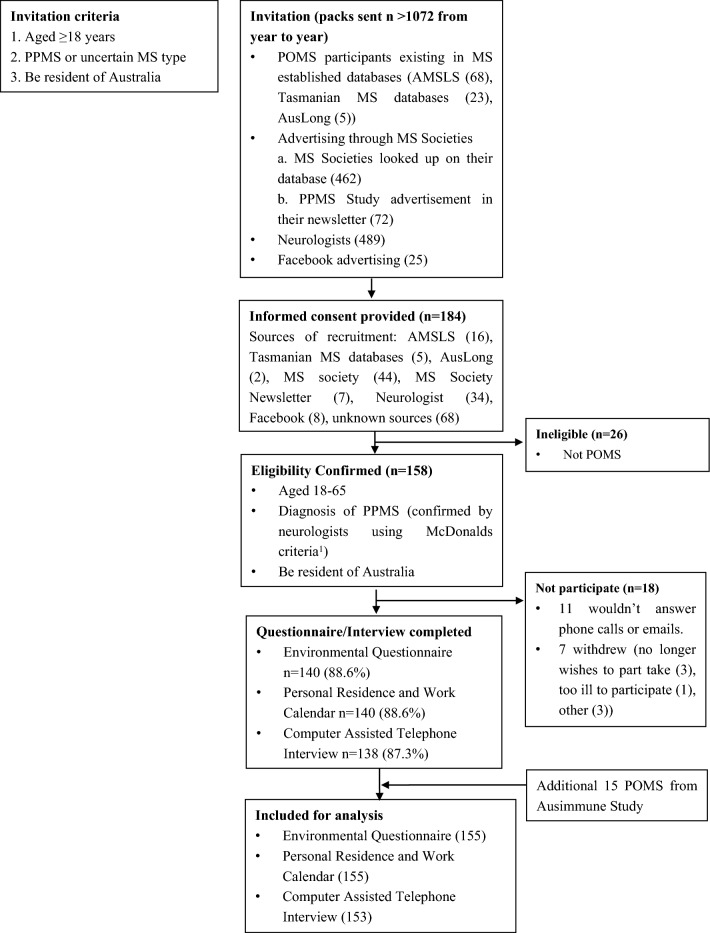
Flowchart for recruitment and participation of POMS participants of PPMS Study from year to year

Figure 1 provides the flowchart of POMS participant recruitment (Oct 2015–May 2019). We used the following methods to recruit POMS participants:

1. From established MS databases, we approached people who met the inclusion criteria, and sent them invitation packs:
  - AMSLS: from the AMSLS, 68 participants were sent invitation packs.
  - Tasmanian MS Longitudinal (MSL) Study: from previous Tasmanian MSL Study, we sent out 23 invitation packs.
  - AusImmune/AusLong Study: from the AusImmune/AusLong Study, we identified five people under age 60 and diagnosis with POMS. We cross-checked the name, date of birth, and sex to ensure they were not duplicates of AMSLS and/or the Tasmanian MSL Study databases and sent out invitation packs.
2. We approached Australian MS Societies from the states and territories of Australia (Western Australia, Queensland, South Australia and Northern Territory, New South Wales, Victoria, Tasmania, and Australia Capital Territory) and asked them to send invitation packs to clients in their databases identified as having POMS. A total of 462 invitation packs were sent by MS Societies. In addition, the MS Societies for New South Wales, Victoria, and the Australia Capital Territory advertised the study in their newsletters. A total of 72 invitation packs were sent to the societies to be sent to their clients who responded to the newsletter advertisement.
3. We contacted 95 Australian neurologists and asked them to send invitation packs to patients in their databases identified as having POMS. A total of 489 invitation packs were sent to the neurologists to be sent to their patients.
4. We recruited participants via Facebook by sponsoring a Facebook post inviting Australian residents over the age of 18 years with POMS to register their details and be sent an invitation pack. The Facebook settings were such that the post would appear in the newsfeed of users that liked MS-related pages or were part of support groups of MS. This subgroup of users would likely have had an interest in MS, have MS themselves, and/or know someone with MS. In addition, Facebook pages with large followings, such as MS Research Australia (> 16,000 followers at the time), MS Australia (> 16,000 followers), Multiple Sclerosis Advisory Council (> 2400 followers), and Kiss Goodbye to MS Australia (> 94,000 followers) shared our recruitment post to increase interaction. A total of 25 invitation packs were sent through this recruitment strategy.

### Participants recruited

From a total of more than 1072 invitation packs sent, 184 (17.2%) provided a statement of informed consent form to participate in this study (Fig. 1). The highest numbers of participants were recruited via the MS Societies (*n* = 51) and neurologists (*n* = 34). Of the 184 people who provided consent, 26 (14.1%) did not fulfill the criteria for POMS, leaving 158 eligible participants. Of the 158 eligible participants, 11 did not return questionnaires after multiple contact attempts (Fig. 1), and another 7 eligible participants withdrew from the study, including 3 who no longer wished to participate, 1 who was too ill to participate, and 3 who had other reasons. A total of 140 participants completed the mailed Environmental Questionnaire and the Personal Residence and Work Calendar (Calendar), and 138 completed the 1-h Computer-Assisted Telephone Interview (CATI). Ten participants completed the CATI survey on paper because they were unwell or had speech difficulties, and five participants completed the CATI over two sessions due to fatigue.

Table 1 shows basic demographic and disease characteristics of the participants. Compared to ROMS cases and their matched controls, POMS cases were more likely to be male, were more likely to be born in an earlier decade and substantially older at interview, were somewhat older at age of first symptom, and were more likely to live between 28.9 and 39.4° S latitude bands. POMS cases also had higher disability scores at the time of interview compared with the ROMS cases. Although the AusImmune POMS cases were incident cases and thus younger in age, with shorter disease duration and milder disability, there were no significant changes in the characteristics when they were combined with the PPMS Study recruited cases.

**Table 1:** Demographic and clinical characteristics of participants in PPMS study, AusImmune study

	PPMS POMS	PPMS and AusImmune POMS	MSBase All POMS	MSBase Australian POMS	AusImmune ROMS	Ausimmune Controls
	(*n* = 140)	(*n* = 155)	(*n* = 4094)	(*n* = 386)	(*n* = 204)	(*n* = 558)
Female, *n* (%)	75 (53.6%)	85 (54.8%)	2146 (52.4%)	222 (57.5%)	166 (81.4%)	436 (78.1%)
Period of birth year, *n* (%)						
Before 1960	49 (35.0%)	59 (38.1%)	1565 (39.1%)	153 (40.6%)	44 (21.6%)	169 (30.5%)
1960–1969	72 (51.4%)	74 (47.7%)	1316 (32.9%)	146 (38.7%)	75 (36.8%)	180 (32.4%)
After 1970	19 (13.6%)	22 (14.2%)	1121 (28.0%)	78 (20.7%)	85 (41.7%)	206 (37.1%)
Year of birth, mean ± SD	1962 ± 5.64	1962 ± 6.41	1964 ± 10.83	1962 ± 9.38	1967 ± 9.53	1965 ± 9.76
Age at first symptom (years), mean ± SD	41.09 ± 8.11	41.44 ± 8.38	38.85 ± 10.11	40.47 ± 9.59	36.97 ± 9.52	–
Age at diagnosis (years), mean ± SD	45.20 ± 7.96	45.15 ± 8.21	42.59 ± 10.25	43.71 ± 9.54	38.92 ± 9.64	–
Age at interview (years), mean ± SD	55.29 ± 5.98	54.50 ± 6.97	49.85 ± 9.92	52.54 ± 8.68	37.73 ± 9.59	39.98 ± 9.75
Disease duration since first symptom (years), mean ± SD	14.25 ± 7.76	13.08 ± 8.20	11.00 ± 7.90	12.08 ± 8.21	0.77 ± 1.03	–
Latitude band^a^						
≤ 28.9 °S	20 (14.3%)	27 (17.4%)	212 (5.18%)	43 (11.1%)	69 (33.8%)	183 (32.8%)
28.9–34.6° S	46 (32.9%)	48 (31.0%)	572 (13.97%)	148 (38.3%)	32 (15.7%)	111 (19.9%)
34.6–39.4° S	51 (36.4%)	56 (36.1%)	948 (23.16%)	172 (44.6%)	49 (24.0%)	148 (26.5%)
> 39.4°S	23 (16.4%)	24 (15.5%)	2364 (57.69%)	23 (6.0%)	54 (26.5%)	116 (20.8%)
Education level^b^						
Primary school or less	4 (2.9%)	5 (3.3%)	276(26.8%)	0	3 (1.5%)	16 (2.9%)
Secondary/technical education	91 (65.5%)	101 (66.0%)	296(28.7%)	34 (45.9%)	148 (73.3%)	395 (71.3%)
University	42 (30.2%)	45 (29.4%)	459(44.52%)	40 (54.1%)	51 (25.3%)	143 (25.8%)
EDSS at interview^c^, mean ± SD	5.77 ± 1.67	5.63 ± 1.76	5.49 ± 1.83	5.72 ± 1.89	1.39 ± 1.24	–
Disability category at interview						
No disability (EDSS < 2)	0	1 (0.7%)	85 (2.5%)	10 (3.6%)	116 (59.8%)	–
Mild disability (EDSS 2–3.5)	22 (16.1%)	29 (19.1%)	554 (16.5%)	38 (13.7%)	70 (36.1%)	–
Moderate disability (EDSS 4–6)	35 (25.6%)	39 (25.7%)	1405 (41.7%)	99 (35.7%)	7 (3.6%)	–
Severe disability (EDSS 6.5–9.5)	80 (58.4%)	83 (54.6%)	1324 (39.3%)	130 (46.9%)	1 (0.5%)	–

Patient characteristics from MSBase were used to assess the representativeness of the recruited POMS participants. PPMS Study: Primary Progressive Multiple Sclerosis Study; AusImmune Study: The Australian Multi-center Study of Environment and Immune Function Study; POMS: progressive-onset multiple sclerosis

^a^In MSBase, latitude of the relevant MS clinic was used as the proxy of latitude of residence

^b^Education data were only partly available for MSBase

^c^For POMS cases, the EDSS was measured by a telephone assessment of EDSS. For ROMS cases, the EDSS was assessed by a neurologist after recruitment into the study. In MSBase, the EDSS was assessed by neurologists during clinic encounters. The demyelinating event that brought them into the study may have resulted in a temporary residual disability

**Fig. 2:**
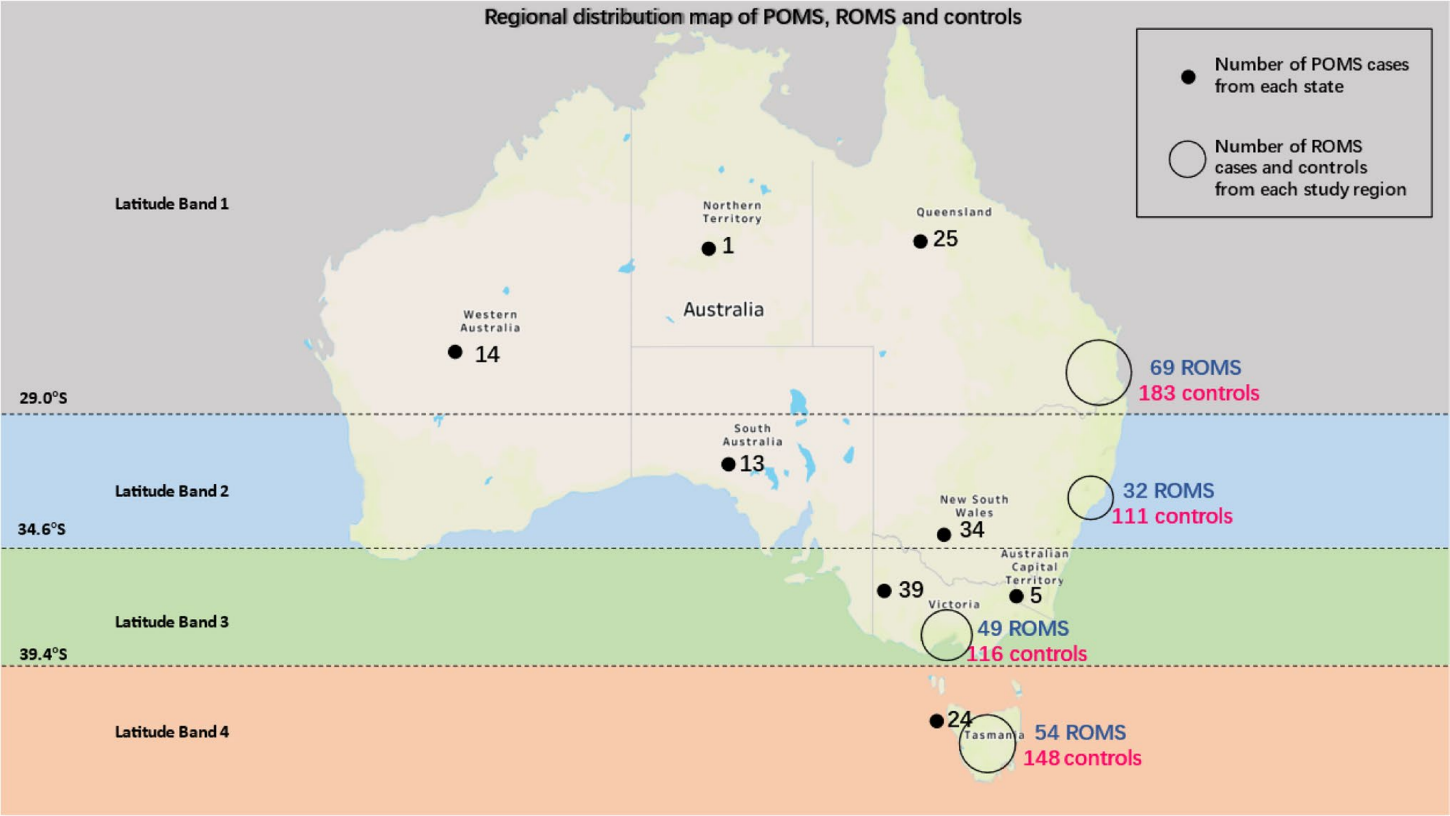
The map of POMS cases from the PPMS Study and ROMS cases, controls from the AusImmune Study^1^. 1. AusImmune Study participants were recruited from four study regions: Brisbane City (latitude 27° South), Newcastle City and surrounds (33° South), Geelong City and the Western Districts of Victoria (37° South), and the island state of Tasmania (43° South)

Figure 2 shows the distribution of the POMS participants and controls within Australia. The majority of POMS participants were located in or near the four AusImmune regions that the ROMS and controls lived. However, there were also groups who lived away from these four regions, including people living in the Australian Capital Territory, South Australia, Western Australia, and in the far north of Queensland.

### Measurements

The data collection was completed between November 2015 and November 2020. Nearly all data collection was completed prior to the 2019 coronavirus (COVID-19) outbreak and the study was not impacted by COVID-19. Participation involved: (1) the completion of a mailed Environmental Questionnaire and a mailed Personal Residence and Work Calendar, and (2) participation in a CATI. The first 48 participants were also asked to visit a local pathology to provide a blood sample.

For the questionnaires, validated measures were used where possible. The participants were interviewed in a standardized manner by a study officer for the CATI. The survey tools and standardized protocol were largely identical to those used in the AusImmune Study [24], the latter reducing interviewer bias and inter-interviewer differences. However, some sections of the questionnaire were removed (current dental health; stressful life events in the previous 12 months), because for prevalent cases, current/recent exposures would not assess the exposure prior to the disease onset. Face-to-face interviews were replaced by CATIs, and instead of the interviewer assessing the most appropriate skin, eye, and hair color from reference charts, this was done by the participants. The CATI additionally assessed disability and the symptoms at onset, which were queried by neurologists in AusImmune Study.

The data collected included demographic details, early-life exposures (breastfeeding, childcare attendance, supplement use, exposure to younger and older siblings), gynecologic/reproductive history (females only), sun exposure history, skin type (color, sensitivity to sun), tobacco and marijuana smoking, medical history including infectious illnesses, family history of medical conditions, occupational and recreational exposure to chemicals and other exposures, disability level and symptoms at onset, and their beliefs about the causes of MS. A full list of variables is provided in Supplementary Table 1.

In the Calendar, participants completed for each year of their lives their location of residence, number and type(s) of pets, schooling or occupation, and days worked per week. This information was used in the CATI to complete leisure time sun exposure, in summer and winter, and occupational sun exposure, with the interviewer guiding participants through the Calendar to identify blocks of time where sun behavior was similar and where it increased or decreased.

### Addressing design choices that may introduce bias

For cost-efficiency, we decided to re-use the data from 558 controls that were collected as part of the AusImmune Study, rather than spending substantial manpower and resource on collecting data from a new control group. It also allowed us to use the AusImmune data to examine the effect sizes of risk factor for those with ROMS and compare them with those with POMS. There are, however, a number of differences that could potentially introduce bias.

#### Prevalent vs. incident POMS cases

The POMS cases that were recruited as part of the PPMS Study were prevalent cases (mean disease duration of 10.13 ± 7.08 years) while the ROMS cases were incident cases from AusImmune, recruited at the time of their first clinical diagnosis of a demyelinating event. It is preferable to use incident cases in a case–control study because the recall or assessment of exposures is less likely to be influenced by their disease or post-onset disease-related changes. Moreover, the POMS cases had a longer time to recall pre-onset events, potentially leading to some greater inaccuracies. In addition, the diagnosis of MS, their current exposures along with their beliefs about the causes of their MS, may have altered their recall of some queried parameters. Therefore, disease-related changes could potentially influence their exposures prior to the onset of MS. In our questionnaires, we focused on exposures that occurred prior to MS symptom onset. We asked both the cases and controls about the extent that they believed that several factors might have caused their MS. Such participant beliefs about the importance of some factors as causes of MS may have led them to better recall or give greater attention to recollection of such parameters than someone who did not think those factors as important. Querying their opinions about factors’ importance to MS enables us to limit analyses for those factors to those who did not think that exposure was an important risk factor (those who are less likely to be biased in their reporting). We have used this method successfully in the past, where we conducted a sensitivity analysis by limiting to those who did not think sun exposure was an important risk factor and finding a similar effect compared to the total sample, which suggested that the main findings were less likely to be biased [28]. We also utilized memorable past events in the Calendar as guideposts to assist with accuracy of sun exposure recall [29]. Using a Calendar method as well as a questionnaire method will also allow us to assess the consistency of findings between measures.

#### Representativeness and matching of controls

In this study, the POMS cases were intended to represent Australian POMS cases between age 18 and 65 years. To assess the external representativeness of the case group, we compared the POMS cases with the POMS participants in MSBase, an international dataset of MS patients collected by participating neurologists with the informed consent of the participants [30]. We selected MSBase participants with POMS aged 18–65 years old. We then captured their data at their first neurologist visit after 2016 or the nearest visit to 2016, the median year of collection in the PPMS Study. Table 1 shows some minor differences between all the included MSBase POMS patients (*n* = 4094) from 131 MS clinics in 40 countries and those MSBase POMS cases from 21 MS clinics in Australia (*n* = 386). Latitude of the relevant MS clinic was used as the proxy of latitude of residence. Education data were only partially available, and the categorization could have varied depending on the global cut points. The distributions of characteristics of the Australian MSBase POMS and our POMS sample were largely similar, although some small not clinically significant differences were observed.

In terms of differences in the PPMS Study POMS cases and controls, the POMS cases could live anywhere in Australia (ranging in latitude from 17 to 43° S), while the AusImmune controls came from four specific regions as per the AusImmune Study (ranging in latitude from 27 to 43° S). In addition, due to the aforementioned recruitment challenges, we extended the included age for the POMS participants to 65 years while the controls were aged 18–59 years old. Importantly, the controls were matched to the original AusImmune cases by age, sex, and location, whereas the new cases would not necessarily have this matching. Indeed, Table 1 shows the different latitude band distributions between POMS and ROMS cases and controls. We will undertake different methods of analysis to address these differences, including adjusting for age of first symptom, sex, and latitude band, and conducting separate analyses using: (1) matched pairs and (2) weights for the controls to reflect the age, sex, and locational distribution of the Australian source population (see below). Furthermore, for exposures that are particularly influenced by latitude (such as sun exposure), we will consider restricting POMS patients to those residing in the same area as ROMS/controls.

#### Potential period-of-birth cohort effects

In general, people born in the same period are more likely to have subsequent life experiences that are similar, by virtue of living in comparable years. Comparing groups that are not born in the same period, and thus belong to different “birth cohorts”, may introduce bias. The data collection of the controls took place between 2003 and 2006 while the data collection of the POMS cases took place between 2015 and 2021. As described, POMS cases were on average 17.6 years older than those with ROMS and 15.3 years older than the controls at time of interview, while the age of first symptoms only differed by 4.5 years between those with POMS and ROMS (Table 1). As we conducted data collection for the PPMS Study on average 12 years later than the data collection of the AusImmune Study, the mean year of birth was on average 5 years different (Table 1).

To assess whether any differences seen between cases and controls, or between POMS and ROMS, could be partly attributable to these period-of-birth cohort effects, we will examine whether exposures are associated with period of birth. If there is an association, we will first adjust for period of birth and see to what extent the effect size will alter. Second, we will conduct a sensitivity analysis by matching POMS cases and controls on age to see whether the result for the matched analysis provides consistent results with our primary analysis. If, for a particular exposure, we still have concerns about residual bias, we will attempt to restrict participants to the same time period (e.g., individuals born between 1960 and 1969) and conduct subgroup analyses to further mitigate this potential bias.

### Statistical analyses

Based on the aforementioned concerns, we will use the following statistical methods in the future case–control analyses and also as a guide for other relevant research.

The primary method of analysis will be binary logistic regression, conducted separately for the POMS vs. controls, and the ROMS vs. controls, and adjusting for age, sex, and latitudinal band of each subject’s residential location. To adjust for age, we will use the age at first MS symptoms for the POMS cases rather than the age of interview, as for the ROMS, the age at interview is equivalent to the age of first symptoms. In this study, we theoretically have a multinomial outcome (control = 1, ROMS case = 2, POMS case = 3), and the preferred method of analysis would be multinomial regression. However, we will not use multinomial logistic regression to report results due to its limitations that multinomial logistic regression estimates relative risk ratios, not odds ratios. It will only be used to test for differences between coefficients of the same covariate at different levels of the multinomial outcome, as a method of assessing whether the relative risk ratios differ between POMS and ROMS.

As a secondary analysis, the controls will be matched where possible to cases based on age (within 5 years), sex and latitude band. This will replicate the matching of controls to ROMS cases in the AusImmune Study, except that the matching by age in the AusImmune Study had been within 2 years. This matching reduced the 155 POMS cases, 204 ROMS cases, and 558 controls to 61 matched POMS–control pairs and 203 matched ROMS–control pairs. The method of analysis will be conditional logistic regression, conducted separately for the POMS–control pairs and the ROMS–control pairs. This will produce two sets of odds ratio estimates of the effect of study factors. Matching on age to within 2 years was not attempted because of the disproportionate reduction in sample size that would have resulted. We can also match POMS and controls based on year of birth (5-year groups) rather than age of first symptom. This will result in 64 matched pairs. This type of matching can be used for exposures where period of birth is of particular concern.

As an additional secondary analysis, the controls will be weighted to reflect the age, sex, and locational distribution of the Australian source population. Age, sex, and residential data on the Australian population (2016 Statistical Area Level 2, SA2) were extracted from the Australian Bureau of Statistics (ABS) website [31] and used in the weighting. We had two controls under the age of 20 and two controls who were 60 years of age by the time interviews were completed. To avoid high statistical weights, we included these in the 20–24 and 55–59 year age groups, respectively. Controls in each of 64 strata (eight 5-year age groups, two sexes, four latitude bands) were assigned equal weights calculated as the population count for that stratum divided by the number of controls in the stratum. The method of analysis will be two separate binary (weighted) logistic regression controlling for age (age at first symptoms for POMS cases, age at interview for ROMS cases or controls), sex, and latitude band.

## Discussion

The total POMS–ROMS–control dataset comprises 155 confirmed POMS participants, 204 ROMS participants, and 558 controls. This is an internationally unique dataset, allowing the examination of a broad spectrum of environmental factors specifically for POMS as well as a comparison of effect sizes between POMS and ROMS. This project will improve the understanding of the etiology of MS for POMS, which may contribute to unravelling the mechanisms of POMS and ultimately lead to the development of novel treatments and interventions.

With the recruitment, we were surprised how difficult it was to recruit sufficient POMS participants. The highest numbers of participants were recruited via the MS Societies (*n* = 51) and neurologists (*n* = 34), where we mailed invitation packages to clients directly, while the recruitment via Facebook resulted in a low return (*n* = 8). There may be a number of reasons for the poor uptake. First, a high level of disability and symptom burden may have prevented them from participating. It is known that people with POMS have, on average, a higher disability level, a higher symptom load, and lower health-related quality of life compared with those with ROMS [32]. In the current dataset, those with POMS had an average EDSS score of 5.7, with more than half participants (54.6%) in the category of severe disability (EDSS 6.5–9.5). Second, we found that 14.1% (26/184) did not fulfill the criteria for POMS and were ineligible to participate. The diagnosis of the onset type is not always straightforward. It is possible that some people with a progressive-onset type may have been missed.

In terms of the external representativeness of the POMS sample, comparisons with MSBase patients showed that our sample is largely representative in demographic and clinical characteristics, and that despite the difficulties with recruitment, we were still able to recruit participants with a higher disability level.

This study highlighted some differences between POMS cases, ROMS cases, and controls related to age, sex, location of residence, and birth cohort. It will be important to take these factors into account. We will, therefore, adjust for age of first symptom, sex, and latitude, birth cohort, and use confirmatory analysis, including a matching approach and an approach by which we use sampling weights to make the controls better represent the Australian population. However, we will still pay attention when reporting any association as there remains a possibility of residual confounding despite the robust adjustments applied.

While we cannot fully eliminate bias associated with using prevalent rather than incident POMS cases, we will only assess exposures that occurred prior to the disease onset and, for some exposures, we will use sensitivity analyses by limiting to those who do not believe the study exposure is a potential risk for MS. This method was successfully used in previous studies [28].

## Conclusion

This report described the conduct of a case–control study of people with POMS and ROMS to examine whether the established risk factors for MS also hold in people with POMS, whether the effect sizes are similar or different compared to people with ROMS, and whether there are risk factors for those with POMS that have not been shown in ROMS. The identification of potential types of bias as well as having methods to minimize them is essential in the generation of valid results that are representative for other MS populations.

### Supplementary Information

Below is the link to the electronic supplementary material.

Supplementary file1 (DOC 56 KB)

Supplementary file2 (XLSX 35 KB)

## Data Availability

The data described in this manuscript were obtained from the Ausimmune/AusLong Study, the PPMS Study and MSBase Foundation. The data can be made available upon reasonable request. Requests to access the Ausimmine/AusLong datasets should be directed to Professor Ingrid van der Mei, ingrid.vandermei@utas.edu.au. Requests to access the MSBase datasets should be directed to info@msbase.org.
