# Supplementary material for: Examining the environmental risk factors of progressive-onset and relapsing-onset multiple sclerosis: recruitment challenges, potential bias, and statistical strategies
Source: J Neurol. 2023 Sep 28;271(1):472–85. doi: 10.1007/s00415-023-11980-z (PMC10770262; doi:10.1007/s00415-023-11980-z)
Supplement: Supplementary file 1 — Supplementary file1 (DOC 56 KB) [file 415_2023_11980_MOESM1_ESM.doc]

Supplementary Table 1. The PPMS Study measurements

| Measurement category | **Description** |
| --- | --- |
| Demographic details | Sex; date of birth and birthplace; current place of residence; highest level of education completed; current employment status, occupation they had for the longest period; ethnicity and ancestral ethnicity. |
| Early life exposures | Breastfeeding (whether breastfed, if “yes” then the duration of breastfeeding); childcare attendance until age 5 years old; supplements used before the age of 15 years (cod liver oil, vitamin D, calcium, and any other vitamins) |
| Siblings | Birth order among siblings; date of birth of each sibling; relation (brother/sister, and full/other); whether lived together before the participant’s 6 years old |
| Number of children and gynecologic/reproductive history | The number of children (including biological children, stepchildren, foster children, adopted children, etc.); each child’s date of birth, sex, relation (natural/other), and whether lived with the participant before the child’s 6 years old.  For females only: age first menstrual period (menarche); number of liveborn children (parity); number of pregnancies that progressed beyond 20 weeks (gravity) |
| Measures related to exposure to ultraviolet radiation (UVR) and skin phenotype | Phone interview:  Amount of time spent outside in summer and winter at 6-10, 11-15,16-20 years age periods (<1, 1-2, 2-3, 3-4, ≥4 hours per day); sun protection behavior in summer at 6-10, 11-15,16-20 years age periods; activities outside in summer compared with peers at 6-10, 11-15,16-20 years age periods; number of lifetime sunburns; eye colour, hair colour, skin color (Fitzpatrick scale^2^), propensity to tan or burn^3^.  Personal Residence and Work Calendar^4^:  For each year of life: amount of time spent outside in summer and winter (<1, 1-2, 2-3, 3-4, ≥4 hours per day) |
| Smoking | Past and current tobacco and marijuana smoking: age started, amount smoked; any periods of quitting. |
| Infectious illness | Whether had the following infections and during which age period (0-5, 6-10, 11-15, 16-20, >20 years old): chicken pox (varicella zoster), measles, German measles (rubella), mumps, whooping cough (pertussis), cold sores (herpes labialis), herpes genitalis, glandular fever (infectious mononucleosis), school sores (impetigo), skin infections and "other illnesses" where participants could list that wasn’t on the list. |
| Medical history | Participants’ personal medical history and the age of onset including: concussion (how many times, age for each concussion, whether it led to a loss of consciousness or loss of memory), bone fractures (how many fractures and how many events that led to fractures), asthma, hay fever, eczema, allergic reaction to food, shingles (herpes zoster), tonsillectomy, adenoidectomy, rheumatoid arthritis, lupus, type 1 diabetes mellitus, cancers (skin, breast, prostate, colorectal, cervical, cervical pap smear abnormality), schizophrenia, chronic fatigue syndrome, osteoporosis, other significant medical conditions |
| Family history | Family history of mother, father, siblings, and grandparents in relation to the following diseases: asthma, hay fever, type 1 diabetes mellitus, multiple sclerosis, cataract, skin cancer (malignant melanoma, basal cell carcinoma, squamous cell carcinoma), breast cancer, prostate cancer, and schizophrenia |
| Occupational and recreational exposure | Occupational and recreational exposure (since 18 years old) and childhood exposure (0-17 years old) to the following: indoor and outdoor pets, paint/varnish, petroleum product, other organic solvent, metals, smoke fumes, fiber glass/resin, wood dust/sawn wood, pesticides, electricity, radioactive radiation/X-ray examination or treatment. |
| Latitude band of location | The participants’ reported current address was geocoded and stored as coordinates of latitude and longitude, and converted to a latitude band:   - Band 1: ≤28.9°S (using the border between the states New South Wales and Queensland) - Band 2: 28.9°S – 34.6°S (including the large cities Sydney and Perth and their metropolitan areas) - Band 3: 34.6°S – 39.4°S (including the large cities Adelaide, Canberra, Melbourne and Geelong) - Band 4: > 39.4°S (including the state Tasmania) |
| Disability and symptoms at onset | EDSS scores were obtained via the telephone interview using the Edmus grading system^5^. |
| Belief on the causes of MS | The cases were asked to rate the perceived importance (decreasing importance from 1 to 4, and 5 is equal to don’t know) of factors that could be potential causes of MS including: smoking, infections, genetic influence, allergy, stress, concussion, exposure to chemicals, lack of exercise, high climatic temperature, low climatic temperature, high past sun exposure, low past sun exposure, high fat diet, low fiber diet, high body weight, low body weight, others |

**References:**

1. Lublin FD, Coetzee T, Cohen JA, Marrie RA, Thompson AJ. The 2013 clinical course descriptors for multiple sclerosis. Neurology 2020;94:1088-1092.

2. Fitzpatrick TB. The validity and practicality of sun-reactive skin types I through VI. Arch Dermatol 1988;124:869-871.

3. Pezic A, Ponsonby A-L, Cameron FJ, et al. Constitutive and Relative Facultative Skin Pigmentation among Victorian Children Including Comparison of Two Visual Skin Charts for Determining Constitutive Melanin Density. Photochem Photobiol 2013;89:714-723.

4. van der Mei IA, Blizzard L, Ponsonby AL, Dwyer T. Validity and reliability of adult recall of past sun exposure in a case-control study of multiple sclerosis. Cancer Epidemiol Biomarkers Prev 2006;15:1538-1544.

5. Lechner-Scott J, Kappos L, Hofman M, et al. Can the Expanded Disability Status Scale be assessed by telephone? Mult Scler 2003;9:154-159.
